# Competition of Photo-Excitation and Photo-Desorption Induced Positive and Negative Photoconductivity Switch in Te Nanowires

**DOI:** 10.3390/nano12213747

**Published:** 2022-10-25

**Authors:** Yanling Yin, Jing Ling, Liushun Wang, Weichang Zhou, Yuehua Peng, Yulan Zhou, Dongsheng Tang

**Affiliations:** Key Laboratory of Low-Dimensional Quantum Structures and Quantum Control of Ministry of Education, Key Laboratory for Matter Microstructure and Function of Hunan Province, School of Physics and Electronics, Synergetic Innovation Center for Quantum Effects and Application, Hunan Normal University, Changsha 410081, China

**Keywords:** tellurium nanowire, positive and negative photoconductivity, water adsorption, photo-desorption

## Abstract

The photocurrent in tellurium nanowire (Te NW) exhibits a subtle influence by many extrinsic factors. Herein, we fabricate Te NW devices and explore their photoresponse properties in detail. It is observed that the current increases greatly at low environmental relative humidity (RH) under light illumination, demonstrating an evident positive photoconductivity (PPC). However, the photocurrent reduces at high RH, yielding a typical negative photoconductivity (NPC). In addition, when exposed to a proper relative humidity, Te NW devices show PPC immediately and then transfer to NPC gradually under illumination, exhibiting the RH sensitive PPC/NPC switch. It is proposed that the competition between photo-excitation and photo-desorption is responsible for this subtle switch of PPC/NPC. On the one hand, the adsorbed water molecules on the surface of Te nanowires, acting as electron acceptors, lead to an increase of conductance, exhibiting the PPC phenomenon. On the other hand, the photo-desorption of water molecules from the surface results in a decreased carrier concentration in the Te nanowires, yielding the NPC phenomenon. The in-depth understanding of such charge transfer processes between the absorbed water molecules and Te nanowires provides an effective route to modulate the carrier densities and control the PPC/NPC switch, which will accelerate the design and application of novel optoelectronic nanodevices.

## 1. Introduction

Negative photoconductivity (NPC), a phenomenon by which the conductivity of materials decreases under illumination, is uncommon and novel. Recently, various materials, such as InAs, AlN, WO_3_, InN, Pb_1−x_Sn_x_Te nanowires, and carbon nanotubes, as well as others thin films and bulk materials, have demonstrated obvious NPC phenomena [1,2,3,4,5,6,7,8,9,10,11]. Tellurium (Te), with a narrow direct band gap of 0.34 eV, is an important P-type semiconductor. In recent years, Te nanowire has been investigated intensively and demonstrates the interesting piezoelectricity, nonlinear optical response, magneto-transport, and chiral spin properties [12,13,14,15]. In contrast, research on the photoconductivity, especially the NPC phenomenon of Te nanomaterials, is limited despite its potential applications in optoelectronic detectors, switches, memory, gas sensors, and so on [16,17,18,19,20,21]. However, although there are some reports on the synthesis and characterization of Te nanowires and nanobelts, these Te nanowire and nanobelt devices only exhibit positive photoconductivity (PPC) [22,23,24,25,26]. To our knowledge, NPC behavior in Te nanowires has yet to be achieved.

In this paper, we explore the effect of laser irradiation and environmental relative humidity on the transport properties of Te nanowire devices. The devices exhibit linear electrical transport characteristics, and the conductivity increases with rising RHs because of water adsorption. The experimental data demonstrate that under light illumination, PPC effect always occurred at lower RHs, while the NPC phenomenon always appeared at higher RHs. Further adjusting the relative humidity and illumination wavelength, it is found that PPC and NPC coexist in the device under proper relative humidity and laser illumination pf a certain wavelength. Based on the experimental data, we propose that the competition between photo-excitation and light-induced desorption of water molecules is responsible for the PPC/NPC phenomena. The PPC and NPC phenomena switch triggered by illumination wavelength and RHs can be applied to design novel photoelectric logic gate devices and nano-optoelectronic sensors.

## 2. Materials and Methods

The tellurium NWs were synthesized by a simple hydrothermal method that is similar to one previously reported [27]. In a typical synthesis process, sodium tellurate (Na_2_TeO_3_, 0.6676 g) and PVP (1 g) were dissolved into deionized water (20 mL) under robust magnetic stirring at room temperature. Hydrazine hydrate (20 mL) and aqueous ammonia solution (8 mL) were then added into the solution. The mixture was stirred continuously to form a translucent solution. Finally, the solution was transferred to a 100 mL Teflon-lined stainless steel autoclave, which was sealed and heated to 180 °C. After maintaining 180 °C for 3.5 h, the autoclave cooled to room temperature naturally. The Te NWs were isolated from the reaction mixture by high-speeded centrifugation. The reaction mixture was firstly centrifuged to obtain the precipitate. Then, the precipitate was dispersed on acetone, alcohol, and deionized water successively, and centrifuged to eliminate the residual source materials. Finally, the purified Te nanowires was dried at 60 °C for further characterizations. The individual Te nanowires were dispersed on Si substrate with thermally oxidized SiO_2_ (300 nm). Au electrodes were defined on top of Te nanowires by the standard photolithography technique and lift-off process.

The morphology and microstructure of the as-prepared product were characterized by scanning electron microscope (SEM, FEI-NOVA-450, Hillsboro, USA) and transmission electron microscopy (TEM, FEI-TecnaiG2-F20,Hillsboro.USA). The crystal phase structure and lattice vibration dynamic were determined by X-ray diffraction (XRD, BRUKER-D8 Discover, Karlsruhe, Germany) and high resolution Raman spectrometer (Horiba JY, HR Evolution, Kyoto, Japan). Photoelectric measurements were conducted on the Lakeshore probe station at room temperature by using semiconductor characterization system (Keithley SCS 4200, Cleveland, USA) under the illumination of different semiconductor lasers (red: 650 nm, green: 532 nm, blue: 450 nm).

## 3. Results and Discussion

**Figure 1:**
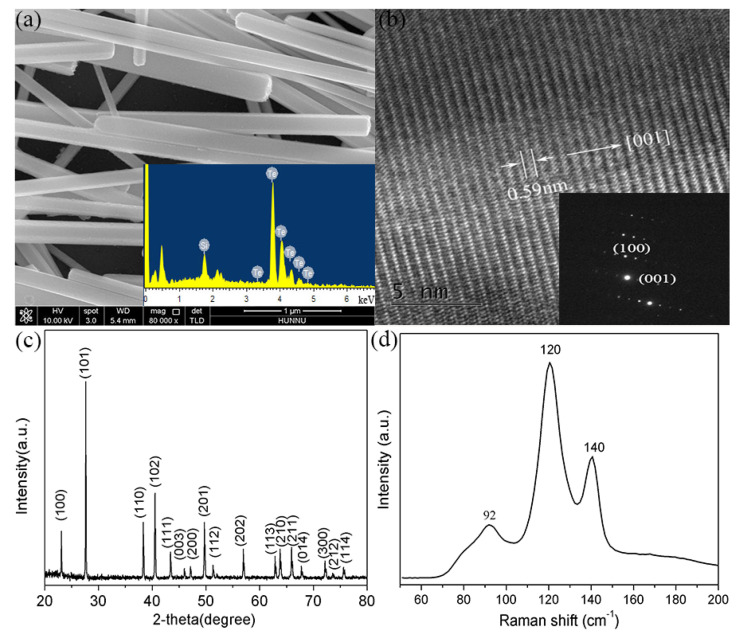
(**a**) SEM of Te NWs. Inset is the corresponding EDS. (**b**) HRTEM of a single Te NW and the corresponding SAED (inset). (**c**) XRD pattern of the as-prepared Te NWs. (**d**) Raman spectrum of Te NWs.

Figure 1a shows the SEM image of as-obtained Te NWs. These Te NWs have a diameter of approximately 200 nm and are several micrometers in length. The energy dispersive spectrum (EDS, inset of Figure 1a) confirms that there is only Te element in the as-synthesized NWs. Figure 1b is the high resolution TEM (HRTEM) image of a single Te NW, from which we can see the clear lattice fringes. The measured fringe spacing of 0.59 nm is in good agreement with the value of (001) plane of hexagonal Te (JCPDS card No. 36−1452), indicating the growth direction along the c-axis of Te NWs with uniform structure and high crystal quality. The corresponding selected area electron diffraction (SAED) pattern further confirms the single-crystalline nature of Te NWs. Figure 1c displays a typical XRD pattern of Te NWs, which can be perfectly indexed and assigned to the hexagonal Te (h−Te) structure (JCPDS card No. 36−1452). Figure 1d is the Raman spectrum of Te NWs used in the following experiments.

**Figure 2:**
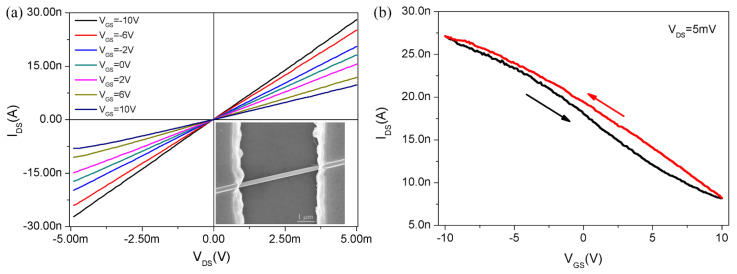
(**a**) Electrical transport characteristic curve (I_DS_−V_DS_) of Te nanowire in vacuum with V_GS_ from −10 V to 10 V. Inset is SEM of the fabricated Te NW device. (**b**) The plot of transfer characteristics at V_DS_ = 5 mV.

The as-prepared Te NWs were dispersed on a Si substrate with oxidized SiO_2_ (300 nm), and the Te NW devices were fabricated by the standard photolithography and lift-off process. The morphology of Te NW devices based on a single Te nanowire is shown in the inset of Figure 2a. Figure 2a shows the electrical transport characteristic curve of a Te nanowire nanodevice in a vacuum (about 10 Pa) with a variable gate voltage (V_GS_) ranging from −10 V to 10 V at a step of 2 V and bias of 5 mV. Obviously, the linear I−V behaviors demonstrate the ideal Ohmic contacts between the Te nanowire and the gold electrodes. Figure 2b is the corresponding transfer characteristic curve. We can see that the drain current (I_DS_) increases (decreases) with decreasing (increasing) V_GS_, consistent with the feature of P-type semiconductor field effect transistors. As is well known, P-type semiconductors are much less than the abundant N-type materials. Thus, the Te nanowires might complement well with the P-type semiconductor family. According to the transfer curve, we calculated hole mobility (*μ*) by using the following formula: μn=dIDSdVGSIn4hdL2πε0εsio2VDS, in which h is the thickness of SiO_2_ dielectrics layer (300 nm), d is the nanowire’s diameter (62.67 nm), L is the active nanowire channel length (4.82 μm), ε_SiO2_ is the dielectric constant of SiO_2_ layer (3.9), ε_0_ is the dielectric constant in a vacuum (8.854 × 10^−12^ F/m), transconductance (dI_DS_/dV_GS_) is −1.23 × 10^−9^ S in the linear regime from −2.5 V to 2.5 V of transfer characteristic curve (I_DS_−V_GS_). After substituting the above parameters, the mobility is determined to be as high as 160 cm^2^ V^−1^ s^−1^ at V_DS_ = 5 mV, which is higher than the previously reported Te nanowires and thin films [28,29]. We also measured the output and transfer curves under a larger bias V_DS_ of 0.1 V, exhibiting the evident linear feature and implying the ohmic contacts. However, the maximum current is high up to 542 nA when a large V_DS_ of 0.1 V is employed, which will bring a great heating effect and can damage the device easily. So, we use a fairly low bias of 5 mV to avoid the influence of the heating effects.

**Figure 3:**
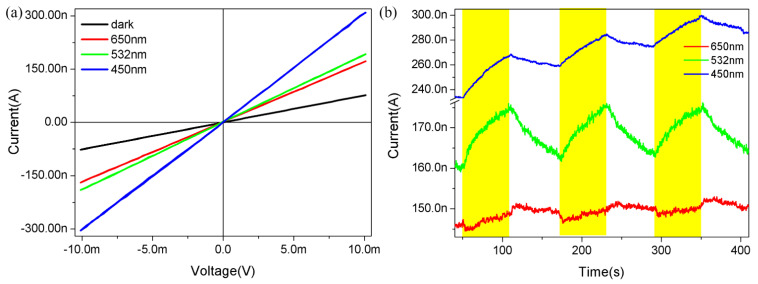
(**a**) Electrical transport characteristics of Te NW devices in dark and under 650 nm, 532 nm, and 450 nm illumination with a relative humidity of 10%. (**b**) The current response curves of Te NW device under 650 nm, 532 nm, and 450 nm illuminations with an applied bias of 10 mV and a relative humidity of 10%. The colored shadow regions represent the periods with the laser turned on.

We then explored the photoresponse behavior of the as-fabricated Te NW devices. In order to avoid the influence of heating effects, we used a fairly low bias and excitation power. Figure 3a illustrates the dark current and photocurrents under 650 nm, 532 nm, and 450 nm laser illuminations, respectively, under an applied bias of 10 mV and relative humidity of 10% in a vacuum. The power of the lasers with wavelengths of 650 nm, 532 nm, and 450 nm was 13.51 mW, 8.25 mW, and 1.63 mW, respectively, measured by a power meter. The power density on the sample of 650 nm, 532 nm, and 450 nm was 4.29 mW/cm^2^, 10.5 mW/cm^2^, and 12.9 mW/cm^2^, respectively. The relative humidity in the vacuum box was regulated by evacuating the air with a mechanical pump and injecting an appropriate amount of warm water. Obviously, the linear I−V curves reveal the ideal Ohmic contact between the Te NW and the gold electrodes, excluding the effect of contact resistance. The dark current was approximately 76.6 nA at the low bias of 10 mV, reflecting the outstanding metal-like conductivity in the semiconductor Te NW. The photocurrent increased to 169.1 nA, 190 nA, and 204.5 nA under laser illuminations of 650 nm, 532 nm, and 450 nm, respectively, owing mainly to the photo-excitation increased carrier density. Figure 3b is the current response curves of the Te NW device under 650 nm, 532 nm, and 450 nm illuminations, respectively, with the applied bias of 10 mV and relative humidity of 10% in a vacuum. The duration of both illuminations (ON state) and no illumination (OFF state) is about 60 s. In Figure 3b, the device current increases with illumination and decreases when the laser is turned off, regardless of the illumination wavelength, exhibiting a continuous and stable positive photoconductivity phenomenon. Under the light illumination, a large number of electrons in the valence band are excited to the conduction band, leaving a large number of holes in the valence band. Owing to the P-type semiconductor feature in the Te NW, the drastic increasing in the concentration of holes will produce the evident positive photoconductivity phenomenon when the Te NW device is applied with an external bias. That is, at low humidity (RH = 10%), the devices show a positive photoconductivity (PPC) behavior. The “ON” state current can also be viewed as containing two components of photo-excitation and photo-desorption. Under the low humidity, the photo-excitation induced current increment is dominant whereas the photo-desorption induced current decrement is minor. The current decrement induced by photo-desorption is equivalent to the slowing down of current increment induced by photo-excitation. A simplified model will be proposed in the later analysis.

**Figure 4:**
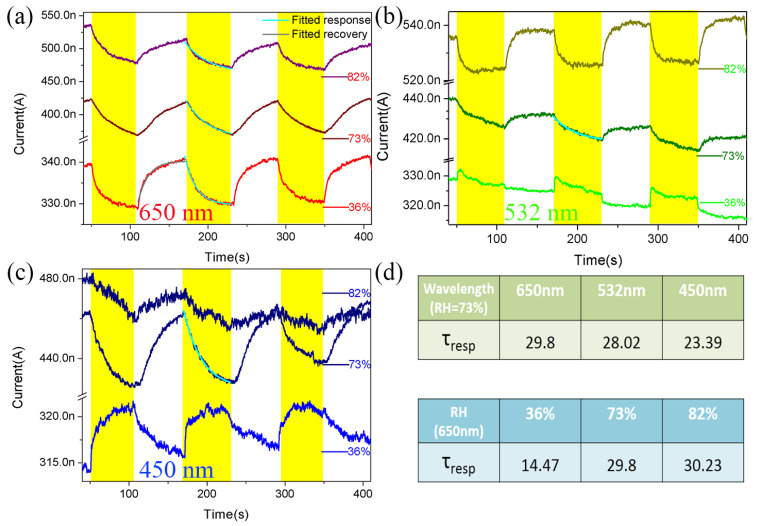
The current response curves of Te NW devices with an applied bias of 10 mV and RHs of 36%, 73%, and 82% under different illumination wavelengths of (**a**) 650 nm, (**b**) 532 nm, and (**c**) 450 nm, respectively. The colored shadow regions represent the periods with the laser turned on. The gray and cyan lines show the corresponding partial fitting curves. (**d**) The green table of $\tau_{resp}$ corresponds to different laser radiations when the ambient RH is 73%, and the blue table of $\tau_{resp}$ under different RHs when irradiated by the 650 nm laser.

We further investigated the photoresponse of Te NW devices in a vacuum box under illuminations of 650 nm, 532 nm, and 450 nm and with relative humidities of 36%, 73%, and 82%. Figure 4a–c are the current response curves of Au/Te NW/Au device under laser illumination and different RH levels. The time period of both illuminations (ON state) and no illumination (OFF state) is about 60 s. A small bias voltage applied to the electrodes is 10 mV. Figure 4a shows the current responses to 650 nm illumination at different RHs of 36%, 73% and 82%. As shown in Figure 4a, the initial dark current increased from 337 nA to 531 nA with RH increasing from 36% to 82%. The data shows that under the same illumination, the device current increases with rising RHs. Figure 4b,c also show a similar trend. The interaction of water molecules with Te is an important process to understand because it can deeply affect the conductivity of the material. Here, water molecules are adsorbed on the surface of Te NWs. Due to their strong electronegativity, the adsorbed water molecules could be ionized by capturing free electrons from Te NWs. At the same time, the induced holes accumulate on Te NWs, resulting in an increase of conductivity of the Te NW devices.

We can see from the bottom curve of Figure 4a, the current drops quickly from 339.5 nA to 328.9 nA under laser illumination (ON state) while it increases to 340.5 nA when the 650 nm laser was turned off (OFF state), demonstrating a strong NPC behavior. The device also exhibits the NPC phenomena at RHs of 73% and 82%, as shown in Figure 4a. Previous reports had suggested that photo-desorption of water molecules was responsible for such laser illumination induced current decreases. [8,24,25,26] During every “ON” cycle, photo-desorption plays a dominant role in photoconductivity and leads to a decrease in carrier density, thereby reducing the current. As shown in the bottom curve of Figure 4b, under 532 nm illumination and a RH of 36%, the phenomena of transient PPC and persistent NPC can be observed in the “ON” cycle, that is, the current suddenly raises sharply and then drops slowly during the “ON” period. When the light is turned on, the current immediately increases from 329.1 nA to 332 nA and then slowly drops to 327.2 nA. Thus, we believe that every “ON” state contains two processes: a fast transient current increment associating with the photo-excitation and a slow persistent current decrement attributing to the photo-desorption. This data provides an empirical evidence to confirm the coexistence and competition between photo-excitation and photo-desorption. The current has a normal and small drop in the “OFF” cycles in the bottom curve of Figure 4b. As shown in the bottom curve of Figure 4c, with 450 nm illumination and under a RH of 36%, the current raises under light illumination and drops in dark. It is observed that the current increases from 314.1 nA to 321.2 nA after irradiation for 60 s while it then decreases to 315.7 nA in the dark. This phenomenon is the normal PPC effect due to the inter-band transition induced by the photo-excitation [4,5,9]. Therefore, the photo-excitation plays a crucial role under 450 nm laser illumination and 36% RH, resulting in the repeatable and stable PPC phenomenon. However, when the RH is increased to 73% and 82%, the device exhibits stable NPC behavior even under 532 nm or 450 nm illuminations, as shown in Figure 4b,c.

From the above experimental data, it can be seen that no matter what wavelength of laser irradiation, with the increase of relative humidity, the PPC behavior of Te nanowire devices in low RH will transfer into the NPC phenomenon. When the device is exposed to high RHs and laser irradiation, photo-generated holes could migrate to the surface of Te NW and neutralize the electrons trapped by the adsorbed water molecules, while the photo-generated electrons can recombine with the holes in Te NWs, resulting in the eventual decreased current and occurrence of NPC in the Te NW devices. Therefore, the NPC in Te NWs is attributed to the influence of strong photo-desorption under high RHs, while PPC at low RH is due to the robust inter-band transition. As a result, there is a competition relationship between the photo-desorption and photo-excitation, yielding the switch between PPC and NPC.

It is known that response time is a key factor that can reflect device performance. To better understand the mechanism of PPC and NPC response intuitively, one complete light switching process and partial response process are fitted with the gray and cyan lines solid lines in Figure 4. The dynamic rise and fall response to a pulse of illumination can be expressed by I = I_0_ + A exp (*t*/*τ*), where I_0_ is the dark current, A is the scaling constant, and *τ* is the time constant related to the response or recovery processes. The time constant can be calculated by fitting the experimental data. Taking the experimental switching cycle curves measured under the 650 nm laser illumination and RH of 36% in Figure 4a for example, the time constants of recovery ($\tau_{rec}$) and response ($\tau_{resp}$) are 13.1 s and 14.47 s, respectively, by fitting the falling and rising edges of a complete light switching process. The time constant (*τ_rec_*) corresponds to the re-adsorption process caused by the removal of the laser, while the time constant $(\tau_{resp}$) relates to the photo-excitation and photo-desorption processes. It is obvious that the time constants for recovery and response are relatively close when the photocurrent exhibits a rapid rise/fall under a typical 650 nm laser pulse and in a RH of 36%. Then, we compared the time constants of response ($\tau_{resp}$) under 650 nm, 532 nm, and 450 nm laser irradiations and in a RH of 73%. It is found that with the decrease of laser wavelength, the time constants $\tau_{resp}$ are 29.8 s, 28.02 s, and 23.39 s, respectively, exhibiting an obvious decrease. At the same time, we also compared the time constants of response ($\tau_{resp}$) in RHs of 36%, 73%, and 82% with a laser illumination wavelength of 650 nm. The time constants are 14.47 s, 29.8 s, and 30.23 s, respectively, with the increase of RH. That is, under the same RH, the shorter the wavelength of the laser, the smaller the time constant of response and under the irradiation of the same laser wavelength, the higher the relative humidity, the larger the time constant of response.

**Figure 5:**
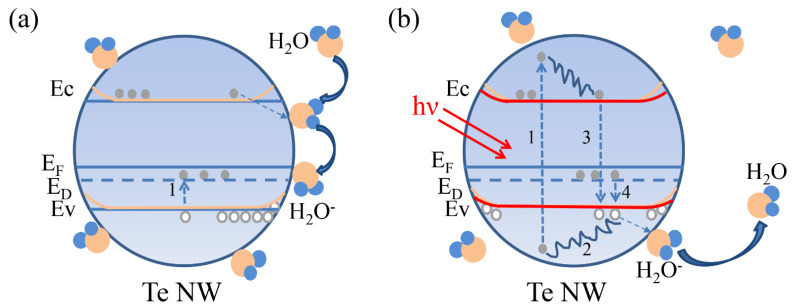
Schematic diagram of energy bands, taking into account the trap level (Ed) and the interaction process of adsorbed water molecules in Te NW (**a**) and light illumination (**b**). The blue lines represent the position of the energy level before H_2_O adsorption, the orange lines represent the energy level after H_2_O adsorption, and the red lines indicate the energy level after light exposure.

From the above results and discussion, a possible simplified mechanism to illustrate the observed PPC/NPC phenomena is proposed. Figure 5 shows the schematic diagram of the PPC/NPC mechanism, which relies on the carrier trapping effect and photo-desorption process induced by the adsorbed water molecules [25,26,30]. In Figure 5a, when the nanodevice is exposed to a humid atmosphere and without illumination, water molecules could be adsorbed on the surface of nanowire. The adsorption process does not lead to structural changes of the material, but it induces P-type doping as electrons can transfer to water molecules and causes the formation of holes inside the Te nanowire. At this time, defect energy level (Ed) is partially filled with electrons that are thermally excited from the valence band, generating more holes in the valence band and resulting in an increase in the conductance of the device. The electrons and the holes are represented by full symbols and open symbols, respectively. Under illumination, as shown in Figure 5b, electrons are excited from the valence band to the conduction band, and then gradually diffuse to the bottom of the conduction band, leaving holes in the valence band (process 1). These holes gradually diffuse to the top of the valence band (process 2). The photo-generated electrons in the conduction band jump down to neutralize the holes in the valence band, causing a drop in the hole concentration (process 3), and electrons from localized states in Ed can recombine with the photo-generated holes in the valence band, reducing the number of holes in the valence band (process 4). That is, under illumination, both the photo-excitation and photo-desorption effect could occur simultaneously. These two effects have a competition relationship. When the relative humidity is lower, there are fewer water molecules adsorbed on the surface of NWs, so the photo-excitation process dominates and the device shows the PPC phenomenon. Under a certain relative humidity and laser wavelength, PPC and NPC coexist in the same device. When the relative humidity is higher, a large number of water molecules are adsorbed on the NWs surface, then the photo-desorption effect is dominant, yielding the evident NPC phenomenon. The device completes the transition from PPC to NPC with increasing RHs.

## 4. Conclusions

In summary, we systematically the photoelectric response of Au/Te NW/Au devices under different illumination wavelengths and RHs. The experimental results indicated that the Te NW presented an excellent and stable NPC effect under high RHs, due to the dominant role of photo-desorption in the competition with photo-excitation during one completed light illumination cycle. In addition, by adjusting the relative humidity and light wavelength, the transfer between PPC and NPC phenomena could be well regulated in Te NW devices. Our study will shed light on the PPC/NPC mechanism of Te NW devices and promote their potential applications in the field of nano-electronics and optoelectronics. In addition, the PPC and NPC switch enables Te nanowires to potentially act as a basic material for the development of sensor devices and nonvolatile memories.

## Data Availability

The data presented in this study are available on request from the corresponding author.
